# Changes in Expression of Key Genes in Ceca of Chicken Broilers as Affected by Glyphosate, Antibiotics and a Coccidiostat

**DOI:** 10.3390/ani14233544

**Published:** 2024-12-08

**Authors:** Georgi Y. Laptev, Daria G. Turina, Vitali Y. Morozov, Elena A. Yildirim, Elena P. Gorfunkel, Larisa A. Ilina, Valentina A. Filippova, Evgeni A. Brazhnik, Natalia I. Novikova, Veronika K. Melikidi, Kseniya A. Sokolova, Ekaterina S. Ponomareva, Vasiliy A. Zaikin, Andrei V. Dubrovin, Peter F. Surai, Darren K. Griffin, Michael N. Romanov

**Affiliations:** 1 BIOTROF+ Ltd., Pushkin, St. Petersburg 196602, Russia; georg-laptev@rambler.ru (G.Y.L.); tiurina2@biotrof.ru (D.G.T.); deniz@biotrof.ru (E.A.Y.); alenkafev@mail.ru (E.P.G.); ilina@biotrof.ru (L.A.I.); filippova@biotrof.ru (V.A.F.); bea@biotrof.ru (E.A.B.); natalia-iv-nov@rambler.ru (N.I.N.); veronika@biotrof.ru (V.K.M.); kalitkina.xeniya@gmail.com (K.A.S.); kate@biotrof.ru (E.S.P.); dfcx@biotrof.ru (V.A.Z.); 2 Federal State Budgetary Educational Institution of Higher Education “St. Petersburg State Agrarian University”, Pushkin, St. Petersburg 196601, Russia; supermoroz@mail.ru; 3 Faculty of Biotechnologies, Information Technologies, Mechanics and Optics (ITMO) University, St. Petersburg 197101, Russia; dubrowin.a.v@yandex.ru; 4 Vitagene and Health Research Centre, Bristol BS4 2RS, UK; psurai@feedfood.co.uk; 5 Faculty of Veterinary Medicine, Trakia University, 6000 Stara Zagora, Bulgaria; 6 Faculty of Agricultural and Environmental Sciences, Szent Istvan University, H-2103 Gödöllo, Hungary; 7 School of Biosciences, University of Kent, Canterbury, Kent CT2 7NZ, UK; d.k.griffin@kent.ac.uk; 8 Animal Genomics and Bioresource Research Unit (AGB Research Unit), Faculty of Science, Kasetsart University, Chatuchak, Bangkok 10900, Thailand; 9 L. K. Ernst Federal Research Centre for Animal Husbandry, Dubrovitsy, Podolsk 142132, Russia

**Keywords:** glyphosate, antibiotic, coccidiostat, gene expression, cecum, broilers, body weight

## Abstract

Studies have shown the presence of residual amounts of the herbicide glyphosate in poultry feed, which leads to its bioaccumulation in the body. Recently, it has been established that exposure to low levels of glyphosate over a long period may have serious negative effects on poultry health. Moreover, combined exposure to several toxicants can potentially lead to additive and/or synergistic effects. The purpose of this study was to analyze changes in meat productivity and the expression dynamics of key genes (*IGF1*, *IGF2*, *MYOG*, *MYOZ2*, *SLC2A1*, *SLC2A2*, *MSTN*, *MUC2*, *OCLN*, *CLDN1*, *TLR2*, *TLR4*, *CAT*, *SOD1*, *PRDX6*, and *HMOX1*) in the cecum of broilers as affected by glyphosate, antibiotics and a coccidiostat (anticoccidial drug). Day-old Ross 308 broiler chickens (*n* = 260) were divided into four groups, including a control group (CONT) fed the basic diet (BD), and three experimental groups: GLY (BD + glyphosate), GLY+ANT (BD + glyphosate and antibiotics enrofloxacin and colistin methanesulfonate), and GLY+CS (BD + glyphosate and the coccidiostat ammonium maduramycin). Samples were collected at control 7, 14, and 40 days of rearing, 50 mg each from three birds from each group. The mean body weight in each group was determined after the individual weighing of the entire flock. At 7 days of age, an upregulating effect on the expression of the immune-related *TLR2* gene was detected in Groups GLY+ANT and GLY+CS compared to Group CONT (*p* = 0.044 and *p* = 0.042, respectively) and Group GLY (*p* = 0.049 and *p* = 0.044, respectively). At 40 days of age, this gene expression, conversely, decreased in Groups GLY+ANT and GLY+CS compared to Group CONT (*p* = 0.041 and *p* = 0.038, respectively). Glyphosate (Group GLY) upregulated the mRNA level of genes associated with productivity (*IGF1*, *IGF2*, and *MSTN*) at 7 days of age by 3.7 times (*p* = 0.041, *p* = 0.036 and *p* = 0.039, respectively) and, conversely, decreased it at a later age (14 and 40 days) compared to Group CONT (*p* = 0.024, *p* = 0.049 and *p* = 0.047, respectively, at 14 days, and *p* = 0.037 and *p* = 0.036 and *p* = 0.035, respectively, at 40 days of age). Thus, we identified detrimental changes in the expression of key broiler genes as influenced by glyphosate, as well as its combinations with antibiotics and a coccidiostat, which may have negative consequences for the poultry industry.

## Simple Summary

It has been established that exposure to trace amounts of the herbicide glyphosate, even in low quantities, may have serious negative consequences for the health of poultry. The purpose of this study was to examine changes in growth and in the expression of key genes in the cecum of broiler chickens after adding glyphosate (a herbicide), antibiotics and an anticoccidial (a drug used to prevent and control infections of intracellular parasites called coccidia) into their food. At 7, 14, and 40 control days of raising, samples were taken. The results showed that at the age of 7 days, there was a stimulating effect on the expression of the *TLR2* gene following exposure to glyphosate, either alone or in combination with antibiotics or an anticoccidial drug. Glyphosate increased the expression of genes *IGF1*, *IGF2*, and *MSTN* associated with broiler performance by 3.7-foldat the age of 7 days and, conversely, decreased the expression of the same ones at later ages (14 and 40 days). Our findings suggest that, in agricultural practice, it is necessary to observe a number of measures to minimize the use of glyphosate and similar compounds that exceed the recommended levels.

## 1. Introduction

A variety of synthetic chemicals, such as herbicides [1], are frequently used to improve the efficiency of modern agricultural systems for raising crops, particularly those used in feed production for farm animals, including poultry [2,3,4,5]. The indiscriminate usage of herbicides has, however, led to widespread concern about the potential adverse effects of these compounds on human and animal health [6,7].

The discovery of glyphosate (GLY; or N-(phosphonomethyl)glycine) by Monsanto was revolutionary for the agricultural industry [1]. As a result, products based on this compound (e.g., RoundUp^®^) have become leading pesticides worldwide [1,8]. Its mechanism of action consists of inhibiting the shikimate pathway for the synthesis of key aromatic amino acids in plants and microorganisms [9]. GLY is effective against more than 100 broadleaf weeds and cereals and more than 60 perennial weeds [7]. The use of GLY has led to the emergence of resistance to it in several dozen weed species [2]. Studies have shown the presence of GLY in poultry feed, which leads to its bioaccumulation in the body [10]. For decades, GLY was considered the safest herbicide used in agriculture [11]. Recent studies have, however, shown that exposure to low levels of GLY over long periods may have serious negative effects on human and animal health [7,12,13,14]. Studies examining the chronic toxicity of GLY in birds have shown its adverse effects on fertility, hatchability and embryonic development, causing reductions in body weight (BW), egg production, shell thickness, egg weight, and chick weight [15].

Combined exposure to multiple potential toxicants, i.e., pesticide residues, antibiotics (ANTs), and anticoccidial agents (or coccidiostats; CSs) can possibly lead to additive and/or synergistic effects [8]. To date, studies assessing chronic exposure to toxicant combinations have been mainly conducted in aquatic species, but only a few have examined this risk in poultry [16]. We previously demonstrated that the dietary supplementation of broilers with GLY, ANTs (enrofloxacin and colistin methanesulfonate), and ammonium maduramicin caused significant changes in the expression of several genes of physiological and economic importance in pancreatic tissue [17]. Around 70% of the avian immune system tissues and organs are located near or within the gut, including the gut-associated lymphoid tissue (GALT), Peyer’s patches, spleen, liver and bursa of Fabricius [18]. In particular, the cecum is of greatest interest for the analysis of immune and productivity gene expression since the cecum microbiome plays a key role in regulating immune function and influencing chicken performance [19]. The cecum is characterized by a high density and complexity of the microbiome, reaching 10^10^ cells per gram. Microbiota in the densely populated cecum provide their host with lactate, acetate, propionate and butyrate through the digestion and fermentation of complex polysaccharides [20]. The profile of these metabolites, altered by a combination of xenobiotics, can have a multifaceted effect on gene expression in this part of the digestive tract.

The purpose of this study was to examine the effects of GLY, two ANTs (enrofloxacin and colistin methanesulfonate) and a CS (anticoccidial drug ammonium maduramycin) by analyzing changes in BW and the expression dynamics of key genes in the cecal tissue of chicken broilers in response to their administration.

## 2. Materials and Methods

### 2.1. Animals, Experimental Design, Diets, and Zootechnical Analyses

The study was carried out in 2023 in a vivarium using a flock of Ross 308 broiler cross broadly exploited in the poultry industry (e.g., [21,22]). The entire experiment lasted until 40 days of age. The chickens were raised in three-tier cages consisting of BB-1 blocks (NPO Stimul-INK, Pushkino, Moscow Oblast, Russia) with free access to water and standard feeds (CJSC Gatchinsky Feedmill, Malye Kolpany, Leningrad Oblast, Russia) as follows: complete starter feed PK5-1G-1101 at 1–4 weeks of age and complete mixed feed PK-6-G-1102 at 28–40 days of age [23]. No special feed additives or therapeutic agents were applied.

In order to avoid errors in testing and accepting hypotheses and to comply with the principles of the humane treatment of laboratory animals, calculations were performed to determine an adequate sample size for the planned experiment [24] using the G*Power program (Version 3.1.9.6; [25]). For this purpose, taking into account the need to identify significant differences between groups with the statistical power (1–β) of 95% and significance level (α) of 5%, as well as with an average expected effect (Cohen’s effect size statistic *f* [26]) of 0.31, the minimum total sample size was determined to be 260 birds.

Day-old chicks (*n* = 260) were randomly divided into four groups (of 65 individuals each): Group CONT, control birds fed the basic diet (BD; *n* = 65); Group GLY, experimental birds fed the BD with the addition of GLY (*n* = 65); Group GLY+ANT, experimental birds fed the BD with GLY and two veterinary ANTs, enrofloxacin and colistin methanesulfonate (*n* = 65); and Group GLY+CS, experimental birds that received the BD mixed with GLY and CS ammonium maduramycin (*n* = 65). Drinking water was supplemented with ANT enrofloxacin as the 10% Enroflon drug solution for oral administration (NPK-VIK LLC, Moscow, Russia) and in the amount of 0.5 mL/L water for the first 10 days of life. ANT colistin methanesulfonate was used in drinking water as the Colistin 2 Million drug (designed by Areal Medical LLC and produced by AVZ-SP, Moscow, Russia) in the amount of 0.25 mL/L water at 33–37 days of age. In the course of this treatment, the birds consumed only water supplemented with these ANTs. Ammonium maduramicin was administered in the amount of 500 g/t feed for the first 35 days of life of chickens. The concentrations of ANTs and CS were selected according to the conventional production scheme and recommendations used in poultry farms in the Russian Federation. The GLY concentration was selected based on the concentration approved as one threshold limit value (1 TLV) for food products (20 mg/kg) [27]. In our previous studies [28], we have shown that the negative effect of GLY begins manifesting in broilers precisely at this concentration.

GLY was used as part of the Agrokiller preparation (CJSC Avgust, Moscow, Russia), containing 500 g/L of GLY acid in the form of isopropylamine salt. The working solution of the preparation was applied to the compound feed by spraying in compliance with personnel safety requirements. The concentration of GLY in feed was monitored by the enzyme-linked immunosorbent assay (ELISA, e.g., [29]) using a Stat Fax 303 Plus photometer (Awareness Technology, Inc., Palm City, FL, USA) and a GLY ELISA Microtiter Plate test system (Eurofins Abraxis, Warminster, PA, USA). The results showed that there were virtually no background traces of GLY in the birds’ diet.

The days of 7, 14, and 40 of bird rearing were set for sampling and weighing. Samples of 50 mg were taken from three birds from each group for gene expression, and the mean BW in each group was determined after the individual weighing of the entire flock. The gain in BW in each group was recorded every week throughout the experiment [23].

### 2.2. Gene Expression Analysis

Total RNA was collected from the cecal tissue of birds three times during the experiment (on Days 7, 14 and 40) by stabilizing the samples with RNAlater (Thermo Fisher Scientific, Inc., Wilmington, DE, USA). The samples were stored at 4 °C overnight (to allow the solution to completely saturate the tissue), and the supernatant was removed and then placed in a −21 °C freezer for storage. The Aurum^TM^ Total RNA mini kit (Bio-Rad Laboratories, Inc., Hercules, CA, USA) was utilized to extract RNA. cDNA was obtained by reverse transcription using iScriptTM Reverse Transcription Supermix (Bio-Rad) and gene-specific primers (Table 1). Primers for the expression analysis were designed using the Primer3 software [30,31] available in Primer-BLAST [32] and Unipro Ugene [33] webtools. The primer specificity and efficiency were repeatedly evaluated in preliminary tests. All primers were rated as highly efficient according to Sreedharan et al. [34]. Amplification was performed using a DTlight amplifier (DNA-Technology, LLC, Moscow, Russia) and the SsoAdvanced^TM^ Universal SYBR^®^ Green Supermix kit (Bio-Rad) in accordance with the manufacturer’s protocol [35]. The following PCR amplification mode and conditions were selected: 5 min at 95 °C (preheating); 30 s at 95 °C, 30 s at 60 °C, and 30 s at 70 °C (40 cycles) [36,37]. Relative gene expression was calculated based on the average expression values of the reference housekeeping beta actin gene (*ACTB*) [38]. All quantitative real-time PCR amplifications were performed in triplicate for each sample and analyzed for differential gene expression using the conventional 2^−ΔΔ*C*^_T_ method [39]. When plotting the respective graphs (bar plots), we were guided by previous similar visual representations in the field of studying differential gene expression (e.g., [40,41]).

### 2.3. Statistical Analyses

Using Microsoft Excel XP/2003 and RStudio (Version 2024.05.0; [42]), a multivariate analysis of variance (multi-factor ANOVA) was implemented to process the results mathematically and statistically by means of the function MANOVA [43] in the bruceR package (version 2024.6; [44]). The analysis results were displayed as the mean (M) and standard errors of the mean (±SEM). Statistically significant differences between groups were accepted at a *p*-value <0.05. The R stats package’s (version 3.6.2; [45]) Tukey’s honest significant difference (TukeyHSD) test and TukeyHSD function [46] were used to compare the means.

## 3. Results

### 3.1. Analyses of Zootechnical Characteristics

The results showed that 7-day-old broilers had an increase in BW by 1.7 g in Group GLY compared to Group CONT (*p* = 0.048; Table 2). This may be a response to the intake of GLY with feed. At later stages of bird growth, an adaptation of the birds’ bodies to this xenobiotic may have occurred.

### 3.2. Alterations in Gene Expression in Cecum

**Figure 1:**
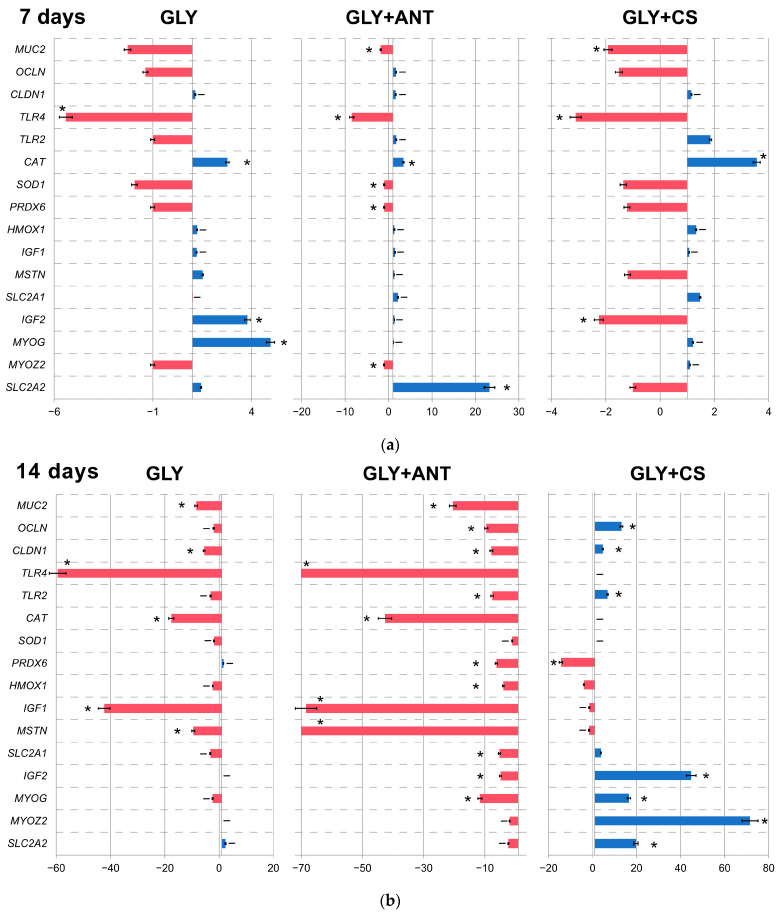

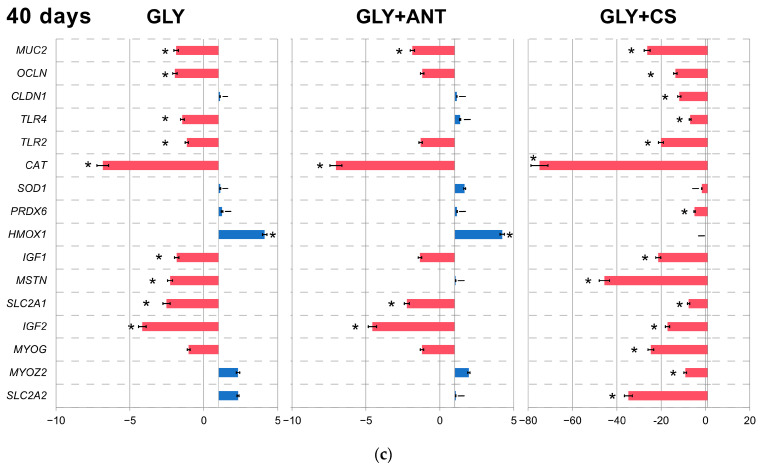
The dynamics of expression levels of key genes in the cecal tissue of Ross 308 broiler chicken groups: (**a**) at the age of 7 days; (**b**) 14 days; and (**c**) 40 days. Red and blue bars on the graphs, respectively, indicate the fold downregulation or upregulation in the level of gene expression in the experimental groups (GLY, GLY+ANT, GLY+CS) relative to Group CONT. * Significant difference compared to Group CONT.

Figure 1 represents the bar plots of the key gene expression dynamics in the cecal tissues of broilers under the influence of xenobiotics. At 7 days of age (Figure 1a), a stimulating effect (i.e., an upregulation by 1.8 and 1.9 times, respectively) on the expression of the *TLR2* gene associated with immunity was noted due to the intake of GLY+ANTs and GLY+CS in comparison with Group CONT (*p* = 0.044 and *p* = 0.042, respectively) and Group GLY (*p* = 0.049 and *p* = 0.044, respectively). In contrast, at 40 days of age (Figure 1c), the expression of this gene was downregulated in Groups GLY+ANT and GLY+CS compared to Group CONT (*p* = 0.041 and *p* = 0.038, respectively). It is noteworthy that GLY alone (Group GLY) and in combination with CS (Group GLY+CS) significantly downregulated the level of the *TLR4* mRNA in chickens at 14 and 40 days of age (*p* = 0.009 and *p* = 0.007, respectively; Figure 1b,c).

We also noted in Group GLY that the mRNA level was upregulated for the genes associated with productivity (*IGF1*, *IGF2* and *MSTN*) by 3.7 times at 7 days of age (*p* = 0.041, *p* = 0.036 and *p* = 0.039, respectively; Figure 1a) and, in contrast, was downregulated at a later age, at 14 (*p* = 0.024, *p* = 0.049 and *p* = 0.047, respectively) and 40 (*p* = 0.037, *p* = 0.036 and *p* = 0.035, respectively) days, compared to Group CONT. At the same time, the expression of the *MYOZ2* and *SLC2A2* genes was upregulated by up to 5.0 (*p* = 0.029) and 2.4 (*p* =0.049) times, respectively, and, in some cases, at different stages of broiler growth compared to Group CONT. In Group GLY+ANT, sudden expression changes in certain productivity genes were also noted. In Group GLY+CS, there was a pronounced upregulation (from 16.5 to 71.5 times) at 14 days of age in the expression of the genes *IGF2*, *MYOG*, *MYOZ2* and *SLC2A2* compared to Group CONT (*p* = 0.001, *p* = 0.002, *p* = 0.001 and *p* = 0.0009, respectively; Figure 1b). At the same time, a sharp reduction in the mRNA of all studied productivity-related genes (i.e., *IGF1*, *IGF2*, *MYOG*, *MYOZ2*, *SLC2A1*, *SLC2A2*, and *MSTN*) was noted at the end of the experiment compared to Group CONT (*p* < 0.05). For instance, the downregulation in the expression level of *SLC2A2* and *MSTN* reached 34.8 and 45.6 times compared to Group CONT (*p* = 0.001 and *p* = 0.0008, respectively).

A stimulating effect (up to 4.2 times) of xenobiotics on the expression of the *SOD1*, *PRDX6* and *HMOX1* genes was noted in Groups GLY and GLY+ANT compared to Group CONT (*p* < 0.05). In Group GLY+CS, a pronounced downregulation of the *SOD1* and *PRDX6* mRNAs (by 1.7 and 4.2 times, respectively) was noted at 40 days of age (*p* = 0.049 and *p* = 0.032, respectively; Figure 1c).

## 4. Discussion

### 4.1. Changes in BW and Expression of Broiler Performance-Related Genes

Quantitative phenotypic traits, such as BW, are controlled by complex sets of genes [47,48,49,50], and differences between fast and slow weight gain in broiler chickens thus depend on both genetic and environmental factors [51,52,53,54]. Herewith, the intestinal mucosa is in almost permanent contact with feed and the toxicants contained within it and directly responds to signals from the intestinal environment [55]. The impact of this interaction on the host can be enormous: from metabolism to the effects on BW [56]. In this study, we observed an increase in BW in 7-day-old birds under the influence of GLY. Previous data also suggest that GLY can increase BW in broilers (e.g., [57]). At the moment, it is unclear by what mechanisms this effect may manifest. We also noted a decrease in the BW of broilers when ANTs were added to feed. It can be assumed that such an effect may be caused by the restructuring of the microbial community in the intestine. For example, it is known that GLY promotes the development of a number of microorganisms that are resistant to it (e.g., [58]). At the same time, ANTs can also affect the numbers of some microorganisms, changing the effect of GLY on BW.

Toxicants in feed may also cause changes in the expression regulation of genes [56], including those that are crucial for growth. For example, the *IGF1* and *IGF2* genes are among the most promising candidate genes for assessing growth performance and carcass quality in chickens [59]. *IGF1* plays an important role in the regulation of skeletal muscle growth during growth and regeneration [60]. *MYOZ2* that belongs to the family of sarcomeric calcineurin-binding proteins is a type of muscle-specific protein that is critical for the growth and formation of skeletal muscles and myocardium [61]. Glucose transport into cells, which is the first rate-limiting step in the regulation of glucose utilization, is mediated by the family of stimulatory glucose transporters (GLUTs) encoded by the SLC2A* genes [62]. It is worth emphasizing that the *MSTN* gene is a member of the transforming growth factor β (TGF-β) family and is also known as growth/differentiation factor 8 (*GDF8*) (e.g., [63,64]). This gene blocks the transcription of genes responsible for myogenesis [65]. The knockdown of myostatin by RNAi demonstrated increased muscle growth in transgenic sheep and chickens [66]. Interestingly, the aforementioned genes, except for *MSTN*, are associated with increased performance [52]. In our study, we noted that GLY upregulated the expression of productivity-related genes (*IGF1*, *IGF2* and *MSTN*) at the age of 7 days (Figure 1a) and, on the contrary, downregulated it at a later age (14 and 40 days; Figure 1b, c). The expression of *MYOZ2* and *SLC2A2* was upregulated, in some cases, at different growth stages of the broilers. A similar picture of some stimulation (increase) in the expression of some productivity-related genes and a decrease in others was observed in the group with the addition of ANT. In Group ANT+CS, a marked upregulation in the expression of *IGF2*, *MYOG*, *MYOZ2* and *SLC2A2* was noted at the age of 14 days (Figure 1b). However, a sharp decrease in the expression of all the studied genes was recorded at the end of the experiment.

The stepwise changes in productivity-relevant gene expression we observed here may be due to the different relative daily growth rates of broiler chickens, which are high at an earlier stage of growth [67]. On average, the BW of broiler chicks at hatching is about 42 g, increasing to 175 g on Day 7 of rearing. The rate of weight increase is 19 g per day (300%) during the first week [67]. The physiological changes that accompany this period are the most significant compared to other stages of ontogenesis. They include the “phenomenal” growth of the digestive system, increased secretion of digestive enzymes, an elevation in the total intestinal surface area used for absorption, improved nutrient transport systems, and the development of the immune system, which is directly related to the level of gene expression [68]. It has been noted that the mRNA expression of some GLUTs, such as *SLC2A8*, peaks at hatching, suggesting a special role of these transporters at the initial stage of bird development, i.e., the period with high energy costs for chicks during pipping and hatching [69].

On the other hand, it has been demonstrated that by Days 15–19 of incubation, bird embryos exhibit the most active transfer of glucose and fatty acids to the neck muscle, which is gradually enriched with glucose, glycogen and protein to maintain incubation activity [70]. The digestion and absorption of nutrients are very complex processes, the components of which exhibit different activity patterns during bird development [71]. In addition, the changing composition of the gut chyme microbiome in birds during individual development can also have different effects on gene expression levels [72]. Indeed, it has been previously shown that aflatoxin exposure, measured by blood aflatoxin–albumin adduct biomarkers, negatively affect *IGF1* expression levels [73].

### 4.2. Genes Associated with the Barrier Function of the Digestive System

The intestinal barrier function is also very important for an organism as it is the first line of defense against pathogenic infection. Tight junction proteins (occludin and claudin) are associated with epithelial cells and act as a barrier, preventing macromolecular translocation [74]. Mucin is an intestinal mucus that plays an important role in protecting epithelial surfaces from pathogens by maintaining colonization with commensal bacteria, a suitable environment for digestion, and facilitating the transport of nutrients from the lumen to the underlying epithelium [75]. Our results showed that, under the influence of chronic xenobiotic exposure, a downregulation (*p* < 0.05) in the expression of the *MUC2*, *OCLN* and *CLDN1* genes was observed in several experimental variants already starting from the 14th day of life (Figure 1b). This may increase intestinal permeability to pathogens. By analogy, *Salmonella* infection also reduced the expression of the *OCLN* and *CLDN1* genes in the ileum and jejunum of broiler chickens and reduced the intestinal barrier function [76].

At 14 days of age (Figure 1b), however, opposite changes in the mRNA levels of the *OCLN* and *CLDN1* genes were noted in Group GLY+CS compared to Group CONT (*p* = 0.049 and *p* = 0.049, respectively). The expression level of these genes was upregulated by 13.1 and 4.7 times, respectively. A decrease in the number of coccidia when using CSs can cause changes in mRNA in host tissues since these parasites, when present in the intestine, can induce changes in the level of the absorption and digestibility of nutrients, enhance mucogenesis, membrane permeability and the availability of nutrients and provoke the proliferation of pathogenic bacteria, which is undoubtedly accompanied by changes in gene expression [77,78,79]. On the other hand, many CSs have been previously shown to disrupt the gut microbiota, which affects gene expression [80]. Lee et al. [81] observed an increase in such cytokine/chemokine transcripts as *CXCLi2*, *IL-4*, *IL-6*, *IL-13*, *IL-17F*, *IFN-γ* and *TGFβ4* (the latter being reassessed as the *TGFB1* gene [82]) in the intestinal epithelium of broilers from groups with the introduction of decoquinate and monensin as CSs in combination with growth-promoting ANTs compared to the untreated Group CONT. Accordingly, we also observed similar effects in our study.

### 4.3. Immunity-Related Genes

The Toll-like receptor (TLR) family is a highly conserved group of genes that are involved in pathogen detection and in the initiation and regulation of innate and adaptive immune responses [83,84], as well as stress tolerance (e.g., [85]). Many factors are known to regulate *TLR* gene expression, including diet composition [86]. The adverse effect of GLY and CS on the expression of some Toll-like receptor genes (e.g., *TLR2* and *TLR4* in our study) may lead to negative consequences. This can occur particularly in industrial poultry farming conditions due to crowding, stress, the presence of pathogen reservoirs, etc. It can lead to an accelerated spread of infectious diseases and a more significant drop in broiler performance than in the more favorable vivarium conditions used in our research.

### 4.4. Genes Associated with Antioxidant Defense

The expression of genes associated with antioxidant defense in Groups GLY and GLY+ANT also changed stepwise during ontogenesis. As known from other studies (e.g., [87]), Hos proteins found in humans, rodents and poultry are involved in induced reactions to oxidative signals. An upregulation in their expression in a body usually indicates the influence of stress factors [88]. For instance, it was previously shown that the mycotoxin citrinin caused the upregulation of several antioxidant genes: *PRDX1* by 1.44 times; glutathione reductase (*GSR*) by 1.78 times; thioredoxin (*TXN*) by 1.25 times; and *TRXRD1* by 3.17 times [88].

The effects of ANTs on gene expression in the broiler cecum observed here may be related to the modulation of the gut microbiome. Previous studies demonstrated that various feed ANTs can enrich the cecum with butyrate-producing bacteria [89,90]. Ruminococcacea abundance was shown to be enhanced by tylosin and enramycin and lowered by salinomycin and monensin [91]. In rodents, intestinal colonization with *Bifidobacterium infantis* or *Faecalibacterium prausnitzii* stimulated the production of Foxp3+ T-regulatory cells and interleukin-10 [92]. Gut microbiota succession over time was associated with different immune gene expression profiles in the ileum [93]. Antimicrobials have been demonstrated to either promote or suppress the levels of cytokine mRNA in human monocytes and neutrophils in vitro [94,95]. These and other studies (e.g., [96,97]) suggest that interventions that alter the quantity or quality of gut bacteria will affect gene expression in gut tissues.

Regarding the effect of GLY on mRNA in birds, previous studies have shown that exposure to triazine herbicides causes oxidative stress, alters antioxidant systems [98,99] and leads to DNA damage [100,101,102]. After 62 days of exposure to low amounts of RoundUp^®^, AMPA (aminomethylphosphonic acid––a primary metabolite of GLY), methylchlorophenoxypropionic acid, acetochlor and 2,4-dichlorophenol, there was an upregulation in the expression of the C1 inhibitor precursor, a crucial negative regulator of the complement system, in European flounders (*Platichthys flesus*) [103]. When exposing common carp (*Cyprinus carpio*) to GLY subacutely for three or seven days, Ma et al. [104] established that there was a significant reduction in the expression of the C3 component and damage to the kidney, which is the principal immune organ in fish. This proved that the complement pathway can be suppressed by GLY. In rats treated with higher concentrations of RoundUp^®^ [105], increased levels of pro-inflammatory cytokines IL-1β, TNF and IL-6, as well as the C-reactive protein in the liver and adipose tissue, were also observed. Previously, we demonstrated that GLY downregulated the expression of antimicrobial and antiviral genes in broilers [21]. Finding further ways to identify and reduce potential risks in the feeding and housing of birds is an important source of increasing the production of broiler chickens [106].

## 5. Conclusions

In the present study, we identified detrimental changes in the expression of such key genes as *TLR2*, *IGF1*, *IGF2*, *MSTN*, *MUC2*, *OCLN* and *CLDN1* as a result of supplementing broilers’ diets with the herbicide GLY, alone and combination with ANTs and CS.

The recent significant increase in the use of GLY has sparked a broad scientific debate about its possible toxicity and potential health effects on humans and animals, including birds. The impact extends not only to organisms that are directly exposed to GLY-containing chemicals but also to those that have no direct contact with this pesticide. Therefore, the assessment of this herbicide in terms of its effect on gene expression in poultry is an important aspect of its toxicity. The importance of gene expression regulation in relation to environmental factors has been increasingly recognized in recent years [107]. In this respect, our findings of detrimental changes in the expression of key broiler genes as influenced by GLY, as well as its combinations with ANTs and CS, may have negative consequences for the poultry industry practice.

Recent results demonstrated that the mechanism of GLY action may involve epigenetic modifications [108]. These are reversible mechanisms associated with tissue-specific gene expression silencing. In particular, GLY has been reported to induce changes in global DNA methylation, specific gene methylation, histone modification, and the differential expression of non-coding RNAs [109]. Therefore, in view of the reported potential risks and based on our own results, we suggest that a number of precautionary measures should be undertaken to minimize the use of GLY and other xenobiotics in an excess of the recommended levels in agricultural practices.

The combined use of GLY and ANTs in agriculture poses a serious threat to the environment. It is believed that their accumulation may harm the diversity and functions of microbial communities, ultimately affecting agricultural productivity as well as human and animal health [110]. There is a concern regarding GLY exposure and the emerging resistance to ANTs in enteric and opportunistic bacteria and pathogenic bacteria [111,112,113]. Also, some researchers have pointed out that GLY can increase cell membrane permeability, which may lead to the increased horizontal transfer of plasmids that promote multidrug resistance to ANTs [114]. Our studies showed that ANTs are able to modify the effect of GLY on both gene expression and ultimately BW.

## Figures and Tables

**Table 1:** Primers for mRNA expression analysis in the cecal tissue of Ross 308 chicken broilers.

Gene and Protein Produced	Forward (F) and Reverse (R) Primer Sequence (5’→3’)
Productivity-related genes	
*IGF1*, insulin like growth factor 1 (Gene ID: 418090)	F: GCTGCCGGCCCAGAA R: ACGAACTGAAGAGCATCAACCA
*IGF2*, insulin like growth factor 2 (Gene ID: 395097)	F: GGCGGCAGGCACCATCA R: CCCGGCAGCAAAAAGTTCAAG
*MYOG*, myogenin (Gene ID: 374004)	F: GGAGAAGCGGAGGCTGAAG R: GCAGAGTGCTGCGTTTCAGA
*MYOZ2*, myozenin 2 (Gene ID: 422682)	F: CAACACTCAGCAACAGAGGC R: GTATGGGCTCTCCACGATTTCT
*SLC2A1*, solute carrier family 1 member 1 (glucose transporter 2) (Gene ID: 396130)	F: AGATGACAGCTCGCCTGATG R: GTCTTCAATCACCTTCTGCGG
*SLC2A2*, solute carrier family 2 member 2 (glucose transporter 2) (Gene ID: 396272)	F: GGAGAAGCACCTCACAGGAA R: CAGGCTGTAACCGTACTGGA
*MSTN*, myostatin (Gene ID: 373964)	F: ATGCAGATCGCGGTTGATC R: GCGTTCTCTGTGGGCTGACT
Genes associated with the barrier function of the digestive system	
*MUC2*, mucin 2, oligomeric mucus/gel-forming (Gene ID: 423101)	F: CTGGCTCCTTGTGGCTCCTC R: AGCTGCATGACTGGAGACAACTG
*OCLN*, occluding (Gene ID: 396026)	F: ACGGCAGCACCTACCTCAA R: GGGCGAAGAAGCAGATGAG
*CLDN1*, claudin 1 (Gene ID: 424910)	F: CATACTCCTGGGTCTGGTTGGT R: GACAGCCATCCGCATCTTCT
Immunity-related genes	
*TLR2*, Toll-like receptor 2 (or *TLR2A*, Toll-like receptor 2A) (Gene ID: 769014)	F: CGCTTAGGAGAGACAATCTGTGAA R: GCCTGTTTTAGGGATTTCAGAGAGATTT
*TLR4*, Toll-like receptor 4 (Gene ID: 417241)	F: AGTCTGAAATTGCTGAGCTCAAAT R: GCGACGTTAAGCCATGGAAG
Genes associated with antioxidant defense	
*CAT*, catalase (Gene ID: 423600)	F: ACCAAGTACTGCAAGGCGAA R: TGAGGGTTCCTCTTCTGGCT
*SOD1*, superoxide dismutase 1, soluble (Gene ID: 395938)	F: CGGGCCAGTAAAGGTTACTGGAA R: TGTTGTCTCCAAATTCATGCACATG
*PRDX6*, peroxiredoxin 6 (Gene ID: 429062)	F: GCATCCGCTTCCACGACTTCCT R: CCGCTCATCCGGGTCCAACAT
*HMOX1*, heme oxygenase 1 (Gene ID: 396287)	F: GGTCCCGAATGAATGCCCTTG R: ACCGTTCTCCTGGCTCTTGG
Gene used as reference control	
*ACTB*, beta actin (Gene ID: 396526)	F: CTGTGCCCATCTATGAAGGCTA R: ATTTCTCTCTCGGCTGTGGTG

**Table 2:** Body weight changes in response to GLY and combinations of GLY, ANTs and CS intake in the four studied groups (CONT, GLY, GLY+ANT, GLY+CS) of Ross 308 broiler chickens.

Body Weight, g	Groups			
	CONT	GLY	GLY+ANT	GLY+CS
at 7 days of age	134.10 ± 7.52	141.20 ± 6.05 *	134.70 ± 7.87	134.50 ± 7.47
at 14 days of age	349.80 ± 19.42	366.00 ± 20.00	352.80 ± 21.63	362.40 ± 38.00
at 21 days of age	756.80 ± 50.10	771.90 ± 63.12	760.70 ± 69.00	767.40 ± 91.02
at 28 days of age	1311.20 ± 74.67	1324.80 ± 82.04	1258.60 ± 97.07	1330.30 ± 69.44
at 35 days of age	2087.40 ± 122.36	2044.90 ± 119.19	2053.70 ± 116.56	2067.90 ± 123.53
at 40 days of age	2567.90 ± 142.90	2514.10 ± 150.78	2506.00 ± 164.11	2556.50 ± 128.48

* Significant difference in Group GLY compared to Group CONT (at *p* = 0.048, as estimated by Student’s *t*-test).

## Data Availability

The original contributions presented in the study are included in the article; further inquiries can be directed to the corresponding author.
